# The longitudinal directional associations of meaningful work with mental well-being – initial findings from an exploratory investigation

**DOI:** 10.1186/s40359-023-01308-x

**Published:** 2023-10-10

**Authors:** Raphael M. Herr, Luisa Brokmeier, Bertil N. Baron, Daniel Mauss, Joachim E. Fischer

**Affiliations:** 1 Center for Preventive Medicine and Digital Health (CPD), Medical Faculty Mannheim, Heidelberg University, Mannheim, Germany; 2 Department of Medical Informatics, Biometry and Epidemiology, Professorship of Epidemiology and Public Health, Friedrich-Alexander-Universität Erlangen-Nürnberg (FAU), Erlangen, Germany

**Keywords:** Meaningful work, Meaning in work, Mental well-being, Purpose, Cross-lagged model, Directional associations

## Abstract

**Background:**

An increasing number of studies reveal that more meaning in life is positively related to mental well-being. Meaning in life can be derived from different sources, including the workplace. The aim of this study was to explore the longitudinal directional association of meaningful work with mental well-being.

**Methods:**

Prospective data from 292 persons at two timepoints (two-week interval) were used to estimate the cross-lagged relationship and directionality of meaningful work with mental well-being.

**Results:**

The cross-lagged panel model had a good fit to the data (Chi^2^ ms(90) = 150.9; p < 0.001; RMSEA = 0.048; p = 0.576; CFI = 0.984; TLI = 0.979; SRMR = 0.040) and showed that levels of meaningful work at t_1_ had a positive effect on mental well-being at t_2_ (β = 0.15, p = 0.010). But mental well-being at t_1_ did not affect meaningful work at t_2_ (β = 0.02, p = 0.652). Sub-analyses revealed the effects to be mainly driven by women (as opposed to men) and white-collar workers (as opposed to blue-collar workers).

**Conclusion:**

This study confirmed a directional association of meaningful work on mental well-being, indicating that more meaningful work has beneficial mental well-being effects.

## Introduction

Growing evidence indicates that the meaning and purpose that persons perceive in life represents an independent determinant of health and well-being (e.g., [1]). Meaning in life is characterized by the extent to which individuals see their lives as having a purpose, a sense of direction, and broader goals to live for [1, 2]. Having a meaning is seen as fundamental to human existence and has been found to be related to a higher ability to handle stress and lower levels of mental discomfort and higher well-being [3–7].

Meaning in life can originate from different sources, including the workplace [6]. “Meaning in work is considered an intrapsychological phenomenon that emerges in the individual’s interaction with his or her working environment. Meaning in work concerns the reasons an individual has for working, what he or she seeks to accomplish by working, and the continuity that he or she experiences in work” ([8]; page 87). Initial empirical findings suggest that meaningful work and mental well-being are interrelated [9, 10]. Meaningful work might increase mental well-being by buffering the impact of work stress and by improving people’s purpose in life [10–12]. The relationship is, however, conceivable in both directions: more meaningful work might lead to better mental well-being, but low mental well-being might also lead to the perception of less meaningful work (e.g., [13]). Thus, longitudinal studies are needed to determine the direction of this relation [10]. Conforming the direction and knowing the strength of the relation can inform about potential interventions to improve mental well-being of employees. For this purpose, this study examines the bidirectional relationships between meaningful work and mental well-being.

It is argued that meaningful work differs in its perceived importance and experience for types of occupation (i.e., white- vs. blue-collar occupations) [14]. Lips-Wiersma and colleagues, for example, report that some aspects of meaningful work were equally important for blue- and white-collar occupations (i.e., unity with others, developing the inner self), while others were more important to white-collar than blue-collar occupations (i.e., expressing full potential and serving others) [14].

In addition to type of occupation, possible variations in meaningfulness can also be expected in terms of gender. Some studies found differences between meaningfulness and gender, but others did not [15–18]. The difference might be related to cultural factors, sample biases, or measurement problems and errors. Overall, the relationship between meaningfulness and gender is not fully understood yet [19]. Therefore it is recommended to take gender into account when researching meaningfulness [20].

Taken together, this exploratory study aims to examine the bidirectional relationships between meaningful work and mental well-being in a longitudinal set-up to generate directions for further research. In addition, potential differences in the effects for type of occupation (white- vs. blue-collar occupations), and gender were tested. This study will thus provide evidence for the contribution of meaningful work to mental well-being of employees and identify groups at risk and ultimately inform about potential preventive interventions.

## Methods

### Study population

The study participants were from a German online panel provided by a commercial service agent. Participants filled out an online questionnaire from 3rd to 8th of December 2018 (t_1_) and again two weeks later (t_2_; 17th to 21st December 2018). The sample was previously quoted (50% female; 50% blue-collar) in order to enable subgroup analyses. Therefore, participants were invited and selected on basis of their initial description until the pursued cell coverage was reached. Several quality checks (e.g., attention check items, filling speed) were carried out and people who did not pass them were excluded. Participants with complete data on relevant variables (i.e., meaningful work and mental well-being) at both time points were include in the analyses (complete cases; n = 292). All participants received an incentive, gave written informed consent and the Ethical Commission of the Medical Faculty Mannheim of the University of Heidelberg approved the study (2018-514N-MA).

#### Meaningful work.

The selection of the items measuring meaningful work was guided by previous research and established questionnaires [21–23]. Accordingly, meaningful work was measured by the following three items: “I enjoy my work”, “My work adds to my sense of purpose in life”, and “I am proud of the work that I do”. All items were rated on a 5-point Likert Scale from 1 = does not apply at all to 5 = fully applies. Cronbach’s alpha was 0.88 at t_1_ and 0.90 at t_2_.

#### Mental well-being.

The five items of the WHO-5 Well-Being Index assessed mental well-being [24, 25]. This questionnaire asks the persons: In the last 2 weeks “… I have felt cheerful and in good spirits”, “… I have felt calm and relaxed”, “… I have felt active and vigorous”, “… I woke up feeling fresh and rested”, and ”…my daily life has been filled with things that interested me”. Cronbach’s alpha was 0.90 at t_1_ and 0.91 at t_2_.

#### Gender and age.

Gender and age were assessed by the standardized questionnaire (men vs. female, age in years).

#### Employment type.

Participants stated whether they were mainly mentally or physically active at work. The former was classified as “white-collar” while the latter were classified as “blue-collar” employees.

### Statistical analyses

First, descriptive analyses were conducted, using univariate analyses with means and standard deviations or number of observations and percentage. Second, zero order correlations of the studied variables were calculated. Third, structural equation modelling tested the cross-lagged relationships between meaningful work and mental well-being using StataSE 14 (College Station, TX: StataCorp LP). A reciprocal model considering forward and reverse relationship between meaningful work and mental well-being was fitted. Estimates based on the maximum likelihood method and measurement errors were allowed to correlate. Model fit was assessed by Chi^2^, the root mean square error of approximation (RMSEA), the Akaike Information Criterion (AIC), the Bayesian Information Criterion (BIC), the Comparative Fit Index (CFI), the Tucker-Lewis Index (TLI), and the standardized root mean squared residual (SRMR). Sub-analyses stratified for gender and employment type (white vs. blue-collar).

## Results

The sample consisted of slightly more women (59%) than men and the average age was 41 years (Table 1). The employment types blue- and white-collar were equally distributed. On an aggregated level, meaningful work did not differ (F_(1,290)_ = 0.87; p = 0.351), but white-collar employees had slightly better levels of mental well-being (mean 2.89 vs. 3.08; F_(1,290)_ = 3.99; p = 0.0467). Women had significantly lower levels of meaningful work (mean 3.42 vs. 3.72; F_(1,290)_ = 7.54; p = 0.0064), and mental well-being (mean 2.84 vs. 3.20; F_(1,290)_ = 14.65; p = 0.0002) than men. There was no association between employment type and gender: 50.8% of men and 51.2% of women were white-collar (χ^2^ = 0.0031; p = 0.954).

**Table 1:** Description of the study population (n = 292)

		n / mean	% / S.D.
Gender			
	Male	120	41.1
	Female	172	58.9
Age (years: range 19–68)		40.80	11.36
Employment type			
	Blue-collar	143	49.0
	White-collar	149	51.0
Meaningful work (range: 1–5)			
	t_1_	3.54	0.95
	t_2_	3.55	0.98
Mental well-being (range: 1–5)			
	t_1_	2.99	0.82
	t_2_	3.07	0.81

The correlation of meaningful work between the two time-points was high (r = 0.858, p < 0.001; Table 2), as it was for mental well-being (r = 0.768, p < 0.001). Meaningful work at baseline was associated with mental well-being at baseline (r = 0.580, p < 0.001), and follow-up (r = 0.551, p < 0.001). Likewise, mental well-being at baseline was related to meaningful work at baseline (r = 0.549, p < 0.001), and follow-up (r = 0.585, p < 0.001).

**Table 2:** Zero order correlations of the studied variables (n = 292)

	Gender (female)	Age (years)	Employment type (white-collar)	Meaningful work t_1_	Meaningful work t_2_	Mental well-being t_1_
Age (years)	-0.322**					
Employment type (white-collar)	0.003	0.033				
Meaningful work t_1_	-0.159*	0.070	0.055			
Meaningful work t_2_	-0.190*	0.095	0.045	0.858**		
Mental well-being t_1_	-0.219**	0.188*	0.117*	0.580**	0.549**	
Mental well-being t_2_	-0.139*	0.081	0.123*	0.551**	0.585**	0.768**

Meaningful work and mental well-being range from 1 to 5

** p < 0.001, * p < 0.05

**Fig. 1:**
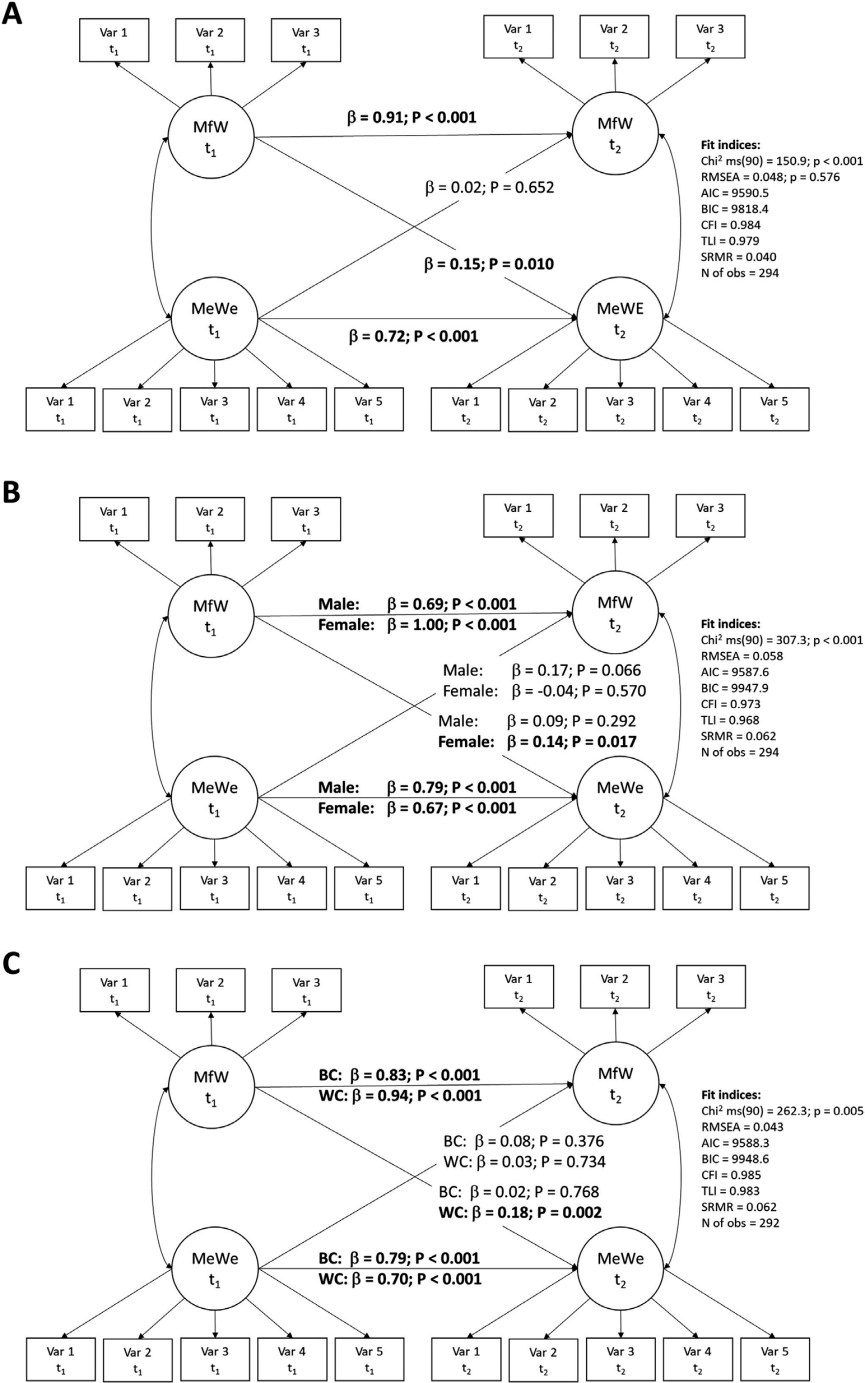
Simplified illustration of reciprocal structural model between meaningful work (MfW) and mental well-being (MeWe). Panel **A**: total sample. Panel **B**: gender stratification. Panel **C**: stratification for white- (WC) and blue-collar (BC) employees. β = Standardized regression coefficients. Significant associations are in bold. Estimates based on the maximum likelihood method. Measurement errors were allowed to correlate to improve model fit

The cross-lagged model revealed a good fit to the data (total sample: Chi^2^ ms(90) = 150.9; p < 0.001; RMSEA = 0.048; p = 0.576; CFI = 0.984; TLI = 0.979; SRMR = 0.040). In the total sample, mental well-being and especially meaningful work were stable over time (β = 0.72; β = 0.91; p-values < 0.001; Fig. 1 Panel A). Meaningful work at t_1_ was associated with mental well-being at t_2_ (β = 0.15; p = 0.010), but mental well-being at t_1_ was not related to meaningful work at t_2_ (β = 0.02; p = 0.652). Stratified analyses revealed that meaningful work had a positive effect on mental well-being in women (β = 0.14; p = 0.017; Fig. 1 Panel B), and white-collar employees (β = 0.18; p = 0.002; Fig. 1 Panel C).

## Discussion

This study revealed that more meaningful work was associated with better mental well-being two weeks later. By contrast, levels of mental well-being were not longitudinally related to meaningful work. These findings can be seen as indication that meaningful work might be a relevant determinant for mental well-being in working populations.

The effect of higher levels of meaningful work on better mental well-being were most evident in women and white-collar employees. In this study, women generally indicated lower levels of mental well-being and meaningful work, though the longitudinal associations of more meaningful work with better mental well-being were especially pronounced with them. While the relationship between meaningfulness and gender might not be fully understood, this study revealed that the relation of meaningful work with mental well-being is evident in women and women might especially benefit of meaningful work [19, 20]. Viewed the other way around, women might have a special risk of mental discomfort if they perceive little meaning in their work. Potential intervention to improve meaningful work might therefore be especially targeted on women.

Differences were also observed regarding employment type. White-collar employees reported better mental well-being and the effects of meaningful work on mental well-being were more pronounced for this group. White- and blue-collar employees did not differ in their ratings of meaningful work. With regard to the finding that the association of more meaningful work with better mental well-being was mainly driven by white-collar workers (as opposed to blue-collar workers), it should be noted that a very rough definition of blue-collar jobs was applied. Further studies might use a finer differentiation between the occupational types.

Although the perception of meaning of work might be generally seen as a relative stable construct, in this study within two weeks – albeit being generally robust (r = 0.858, Table 2) – some variability in its experience was observed; related to changes in mental well-being as well. Accordingly, it may be assumed that some short-time dynamics and within-individual fluctuations in the experience of the meaning of work exists. Thus, what applies to perceived fairness in the workplace (i.e., organizational justice) may also apply to perceived meaning of work. Matta and colleagues report that the fairness perception at work vary substantially on a daily basis [26, 27]. According to our findings, day-to-day variations in meaning of work might also exist. Further studies focusing on the variability of meaning of work are needed to verify this assumption.

Three pathways are assumed to link meaningfulness with health and well-being [28]. First, meaningfulness might enhance psychological and social resources that buffer against stress effects, because people with a higher meaning in life might perceive stressors as less difficult or might be less reactive to stressors. People with a higher meaning might thus be less likely to activate the stress-linked neurohormonal cascade. The second pathway refers to behaviors. Based on the assumption of Victor Frankl [29], that more meaning in life provides persons with a greater will to live, people might engage in more restorative health behaviors, like physical activity or the use of preventive health care, and the attempt to avoid harmful behaviors. Meaning in life might also directly influence biological processes related to health and well-being. This third pathway comprises biological aspects like inflammation, cardiac autonomic function, and biological risk factors like the metabolic syndrome [30], and allostatic load [31]. While the concrete pathway by which a higher meaning in life might affect health and well-being positively appears complex and multifactorial, the effect is evident [28, 32].

In light of this study’s findings that more meaningful work is related to better mental well-being, the question is what can enhance meaningful work? A recent study has shown that general meaning in life can be increased by mindfulness interventions in a sample of women [33]. Thus, mindfulness interventions at the workplace might be beneficial. Regarding meaningful work, Ehresmann and Badura [34] surveyed hospital employees and identified the quality of leadership, the company culture, and the quality of the personal relationships among the employees as main sources of meaningful work. Albrecht and colleagues [35] found in their study the job resources: job variety, development opportunities, and autonomy to be related to meaningful work, with job variety having the strongest correlation. Based on their literature review, Lysova and colleagues [36] recommend organizations to build and maintain work environments that provide opportunities for job crafting by offering well-designed, good-fitting, and quality jobs to enhance meaningfulness at work. In addition, supportive leaders, cultures, policies and practices, and high-quality relationships, as well as access to decent work should have beneficial effects for fostering meaningful work. Meaning in work might, however, not only be determined by work conditions alone, but also by the individual’s ability to recognize and act on what is meaningful and what is not [14, 29]. Taken together, several factors at different levels (individual, job, organizational, and societal) were identified as sources or conducive factors for meaningful work [36]. However, hitherto little can be said about the relative and independent relevance of these aspects or their interactive effects.

While this study adds further knowledge by providing evidence of the positive effects of meaningful work for mental well-being, some limitations must also be considered. The study was accomplished online, which poses the risk of studying a specific sample and the generalization to other populations cannot be taken for granted. Furthermore, the follow-up was set to two weeks and future studies might look at the effects at differing time periods and at multiple measurement points to study the variability of meaning of work. Another aspect refers to the measurement of meaningful work. The complex and multidimensional construct of meaningful work was measured by only three items adapted from different scales. Further studies should apply more comprehensive questionnaires which might also make it possible to examine its sub-dimensions (e.g., significance, broader purpose, and self-realization [37]). Another limitation refers to the measurement of type of occupation, which was rather rough in this study: participants indicated whether they were mainly mentally or physically active at work. As type of occupation seems a relevant factor for mental well-being and the effects of meaningful work, further studies with finer graduation seems therefore necessary.

In conclusion, the present study highlights the initial importance of meaningful work for mental well-being, especially for women and white-collar occupations. Future research and interventions should therefore consider meaningful work as a promising point of leverage to enhance mental well-being of employees.

## Data Availability

The datasets generated and/or analysed during the current study are not publicly available due to German data protection regulations and the assurances in the informed consent agreement and ethic approval that the data will not be disclosed, but are available from the corresponding author on reasonable request.

## List of abbreviations

AIC: Akaike Information Criterion
BIC: Bayesian Information Criterion
BC: blue-collar
CFI: Comparative Fit Index
MeWe: mental well-being
MfW: meaningful work
RMSEA: root mean square error of approximation
SRMR: standardized root mean squared residual
TLI: Tucker-Lewis Index
WC: white-collar
