# Correction: The longitudinal directional associations of meaningful work with mental well-being – initial findings from an exploratory investigation

**DOI:** 10.1186/s40359-023-01408-8

**Published:** 2023-11-07

**Authors:** Raphael M. Herr, Luisa Brokmeier, Bertil N. Baron, Daniel Mauss, Joachim E. Fischer

**Affiliations:** 1 Center for Preventive Medicine and Digital Health (CPD), Medical Faculty Mannheim, Heidelberg University, Mannheim, Germany; 2 Department of Medical Informatics, Biometry and Epidemiology, Professorship of Epidemiology and Public Health, Friedrich-Alexander-Universität Erlangen-Nürnberg (FAU), Erlangen, Germany

**Correction: BMC Psychol 11, 325 (2023)**

**https://doi.org/10.1186/s40359-023-01308-x**

Following publication of the original article [1], the authors brought two errors to the attention of the journal: panel 'c' was missing from Fig. 1; shared senior authorship of the last two authors, Daniel Mauss and Joachim E. Fischer, had been omitted from the author list. The article has since been updated to correct these errors, and the corrected figure and authorship can be seen in this erratum. The journal and the authors thank you for reading this erratum.

**Fig. 1:**
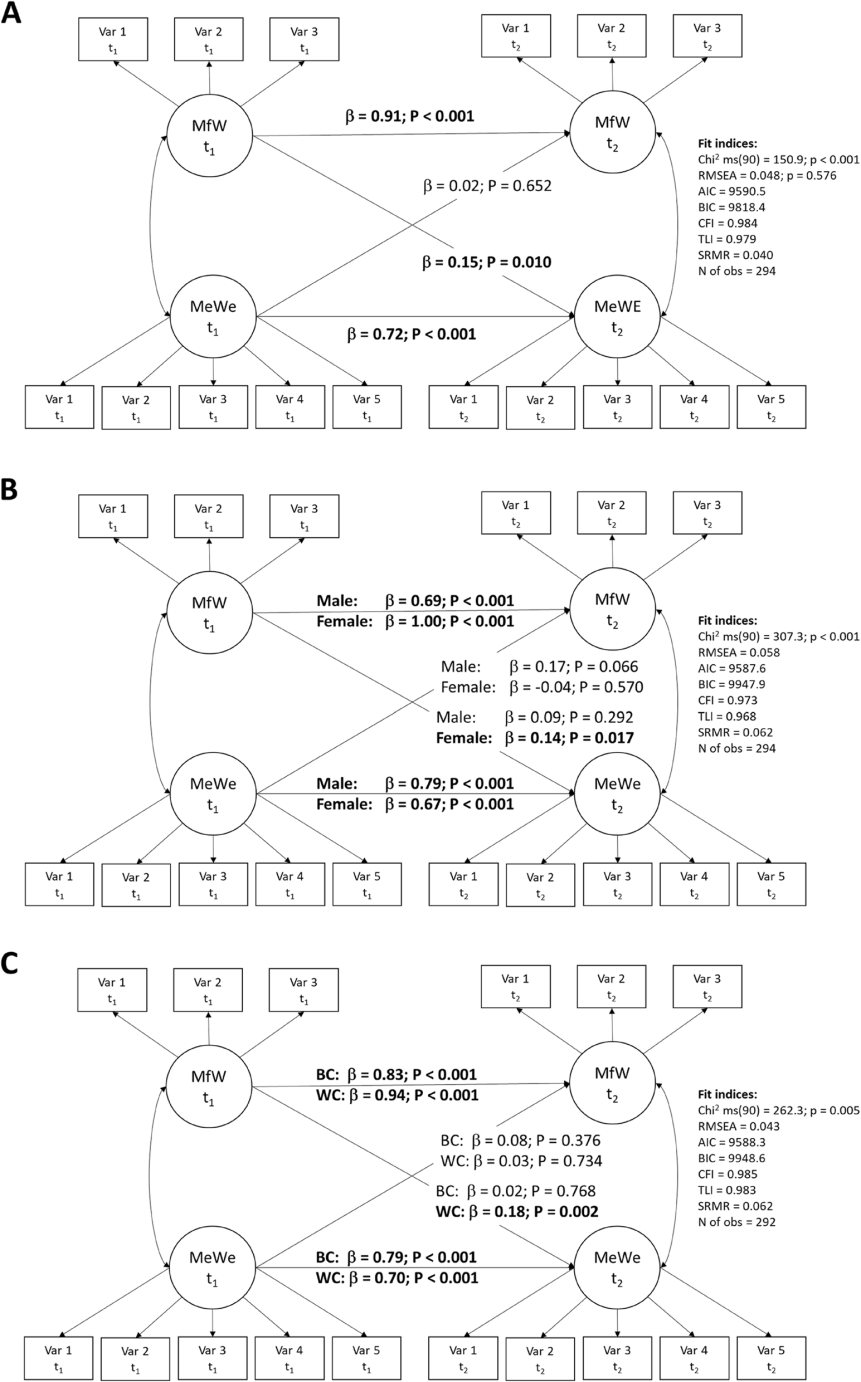
Simplified illustration of reciprocal structural model between meaningful work (MfW) and mental well-being (MeWe). Panel **A**: total sample. Panel **B**: gender stratification. Panel **C**: stratification for white- (WC) and blue-collar (BC) employees. β = Standardized regression coefficients. Significant associations are in bold. Estimates based on the maximum likelihood method. Measurement errors were allowed to correlate to improve model fit
